# MiR-103a promotes tumour growth and influences glucose metabolism in hepatocellular carcinoma

**DOI:** 10.1038/s41419-021-03905-3

**Published:** 2021-06-15

**Authors:** Yuling Liu, Yuanzhou Zhang, Bowen Xiao, Ning Tang, Jingying Hu, Shunshun Liang, Yechun Pang, Huili Xu, Junping Ao, Juan Yang, Xiaofei Liang, Lin Wei, Yunfeng Wang, Xiaoying Luo

**Affiliations:** 1 State Key Laboratory of Oncogenes and Related Genes, Shanghai Cancer Institute, Renji Hospital, Shanghai Jiao Tong University School of Medicine, Shanghai, 200032 People’s Republic of China; 2 Department of General Surgery, Pudong New Area People’s Hospital, Shanghai University of Medicine & Health Sciences, Shanghai, China

**Keywords:** Cancer metabolism, Liver cancer

## Abstract

Hepatocellular carcinoma (HCC) is a common and high-mortality cancer worldwide. Numerous microRNAs have crucial roles in the progression of different cancers. However, identifying the important microRNAs and the target biological function of the microRNA in HCC progression is difficult. In this study, we selected highly expressed microRNAs with different read counts as candidate microRNAs and then tested whether the microRNAs were differentially expressed in HCC tumour tissues, and we found that their expression was related to the HCC prognosis. Then, we investigated the effects of microRNAs on the cell growth and mobility of HCC using a real-time cell analyser (RTCA), colony formation assay and subcutaneous xenograft models. We further used deep-sequencing technology and bioinformatic analyses to evaluate the main functions of the microRNAs. We found that miR-103a was one of the most highly expressed microRNAs in HCC tissues and that it was upregulated in HCC tissue compared with the controls. In addition, high miR-103a expression was associated with poor patient prognosis, and its overexpression promoted HCC cell growth and mobility. A functional enrichment analysis showed that miR-103a mainly promoted glucose metabolism and inhibited cell death. We validated this analysis, and the data showed that miR-103a promoted glucose metabolism-likely function and directly inhibited cell death via ATP11A and EIF5. Therefore, our study revealed that miR-103a may act as a key mediator in HCC progression.

## Introduction

Hepatocellular carcinoma (HCC) is one of the most common cancers worldwide [1] and remains the third leading cause of cancer mortality [2–5]. Although great improvements have been made in diagnostic and surgical techniques and the development of new molecularly targeted drugs, the 5-year overall survival rate is still low [4]. In addition, some patients progress to advanced stages of the disease due to recurrence and metastasis [6, 7]. Therefore, it is necessary to gain a full understanding of the molecular mechanisms underlying the tumorigenesis and tumour progression of HCC.

MicroRNAs (miRNAs/miRs) are a class of small noncoding RNA molecules that are ~22 nucleotides in length [8–10] and regulate gene expression and cellular processes by targeting messenger RNA (mRNA) transcripts [11]. MicroRNAs take part in many important biological processes, including early embryonic development [12] and fat metabolism [13], and they even regulate the differentiation of stem cells [14]. However, the abnormal expression of miRNAs is closely related to the occurrence and development of human cancer (cell proliferation [15], apoptosis [16], and cell death [17]). MicroRNAs have been shown to play crucial roles in the development and progression of different kinds of cancer, including HCC [18–20]. For example, miR-29c-3p acts as a tumour suppressor in HCC by targeting DNMT3B and the LATS1-associated Hippo signalling pathway [21]. MiR-541 potentiates the response of human HCC to sorafenib treatment by inhibiting autophagy [22]. A recent study also indicated that miR-1269b downregulated SVEP1 expression and promoted HCC proliferation and metastasis, likely through the PI3K/Akt signalling pathway [23]. In addition, previous studies have shown that many abnormally expressed microRNAs are closely associated with the prognosis of HCC patients [6, 24, 25]. However, these analyses are mainly based on the results of the differential expression between tumour tissues and normal tissues, whereas the expression level of microRNA is less frequently considered.

The rapid development of high-throughput sequencing technologies and comprehensive bioinformatics analyses allows for the analysis of important differentially regulated molecules in HCC progression and their major functions [26]. Although many studies have focused on differential molecular regulation in HCC progression and its function [27, 28], the main regulatory factors or the main nodes in the regulatory network remain issues that need to be considered. To address this challenge, we analysed HCC microRNA expression data acquired from The Cancer Genome Atlas (TCGA) database. We screened for differentially expressed microRNAs (DEMs) in tumours vs adjacent normal tissues based on the expression level of microRNAs. We hypothesize that these high-expression DEMs have significant functions in HCC progression, and investigations of these high-expression DEMS may obtain information about the main factors or nodes in the regulatory network for HCC progression.

In the present study, we found that miR-103a is a highly expressed and upregulated microRNA in HCC tissues and showed that its high expression is associated with poor prognosis in patients with HCC. We further found that miR-103 promotes cell proliferation, migration, invasion and glucose metabolism in vitro and that miR-103a promotes tumour growth in vivo. Glucose metabolism is the main function of miR-103a. Our study suggested that high expression and upregulation of miR-103a are the main differentiation factors in the regulatory network and indicated that the ability of miR-103a to promote glucose metabolism function might represent the main change in HCC progression.

## Results

### miR-103a is a highly expressed and upregulated microRNA in HCC tissues

**Fig. 1:**
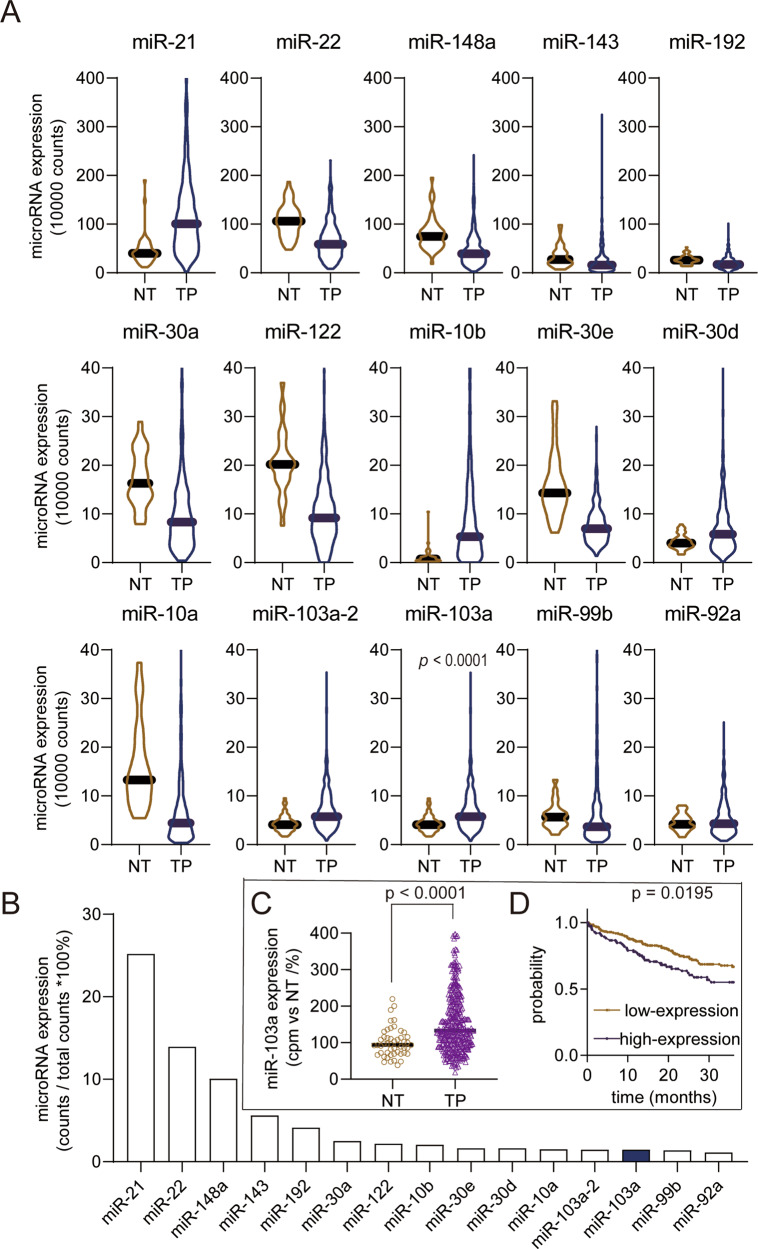
miR-103a is a highly expressed/upregulated microRNA in HCC, and its high expression is related to poor prognosis in TCGA datasets. **A** Fifteen highest microRNA read counts in hepatocellular carcinoma tissues (*n* = 372) and adjacent normal tissues (*n* = 50). **B** MiR-103a reads in next-generation sequencing accounted for 1.458% of the total reads, and miR-103a was the 13th most highly expressed microRNA in HCC tissues. **C** miR-103a expression levels (normalized reads) were upregulated in HCC tissues (*n* = 372) compared with adjacent normal tissues (*n* = 50). **D** Kaplan–Meier analysis showing that the level of miR-103a was negatively correlated with the overall survival rate of HCC patients (follow-up threshold was 36 months). NT adjacent tumour tissues, TP tumour tissues. miR-103a microRNA-103a, HCC hepatocellular carcinoma cells. These data were obtained from TCGA database.

We performed an expression analysis of publicly available microRNA sequencing data from HCC patients in the TCGA database. Then, microRNAs were sorted from high to low according to the read counts in HCC tissues. We identified 15 top-ranked microRNAs (Fig. 1A), and 6 were upregulated while 9 were downregulated (Fig. 1A, according to the read counts, not normalized). Furthermore, we detected the microRNA proportion compared to the total microRNA sequencing read counts in HCC tissues, and the data showed that the miR-103a read counts were 1.458% of the total microRNA read counts (Fig. 1B). The above data showed that miR-103a was a high-expression microRNA. miR-103a plays a vital role in physiological [29] and pathological processes [30]. Because of the limited studies of liver cancer, we chose this microRNA as a candidate.

High-expression/differentiated expression microRNAs might play an important role in HCC progression. To validate that miR-103a is a DEM in HCC, we checked the miR-103a expression in TCGA datasets after normalizing the read counts. The data showed that miR-103a expression was 1.6-fold upregulated in HCC tissues (*n* = 372) compared with normal adjacent tissues (*n* = 50) (Fig. 1C, *p* < 0.0001, unpaired). High expression of miR-103a was associated with poor prognosis of HCC patients in TCGA (Fig. 1D, follow-up cut-off is 36 months).

**Fig. 2:**
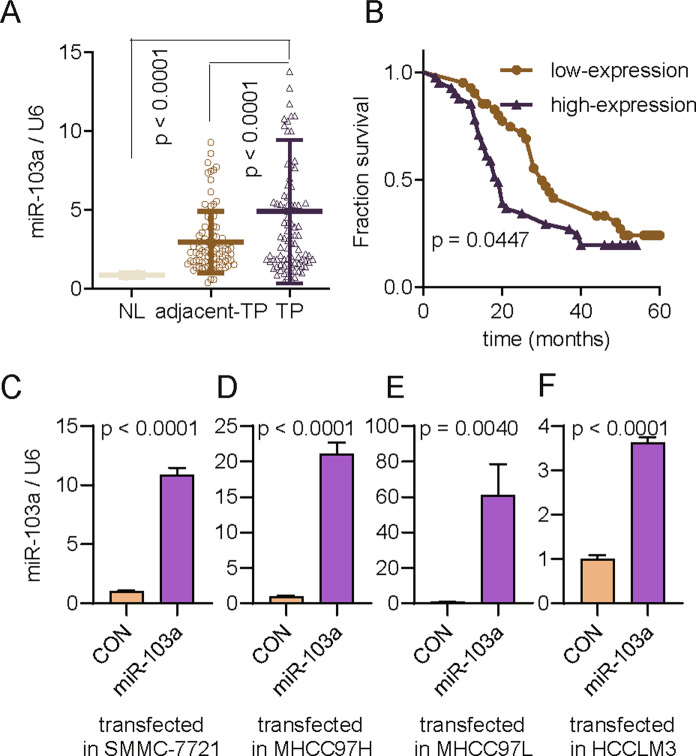
miR-103a expression was upregulated in HCC and related to poor prognosis in the Renji cohort. **A** Reverse transcription-quantitative PCR analysis was performed to assess miR-103a expression in 89 paired HCC tissues and adjacent normal tissues and two healthy volunteer liver tissues. **B** Survival curves suggested that increased miR-103a expression was significantly associated with unfavourable prognosis of patients with HCC. **C**–**F** Reverse transcription-quantitative PCR analysis was performed to validate miR-103a expression in SMMC-7721 (*p* < 0.001), MHCC97H (*p* < 0.001), MHCC97L (*p* < 0.01) and HCCLM3 (*p* < 0.001) cells transfected with miR-103a lentivirus. All results are representative of at least three independent experiments.

Alternatively, miR-103a levels in 89 primary HCC and paired adjacent tissue samples were evaluated by real-time PCR (Table S1)Then, we checked the expression of miR-103a in our cohort, and the data showed that miR-103a was 1.94-fold upregulated in HCC tissues compared with adjacent tissues (Fig. 2A, *p* < 0.0001, paired *t* test, *n* = 89). To evaluate the potential prognostic value of miR-103a in HCC, we performed an overall survival analysis of our cohort, and the data showed that the upregulation of miR-103a in HCC patients was correlated with poorer survival (Fig. 2B, *p* = 0.0447). As expected, we obtained similar results in our cohort as the TCGA database analysis.

These results showed that miR-103a is a highly expressed and upregulated microRNA in HCC tissues, and its high expression is associated with poor prognosis.

### miR-103a promotes the proliferation of HCC cells in vitro

To further investigate the biological function of miR-103a in HCC, four types of HCC cells (SMMC-7721, MHCC97L, MHCC97H and HCCLM3), which are widely used in the study of HCC, were transfected with lentivirus-miR-103a or lentivirus-control, and the transfection efficiency was determined via qRT-PCR analysis (Fig. 2C–E).

**Fig. 3:**
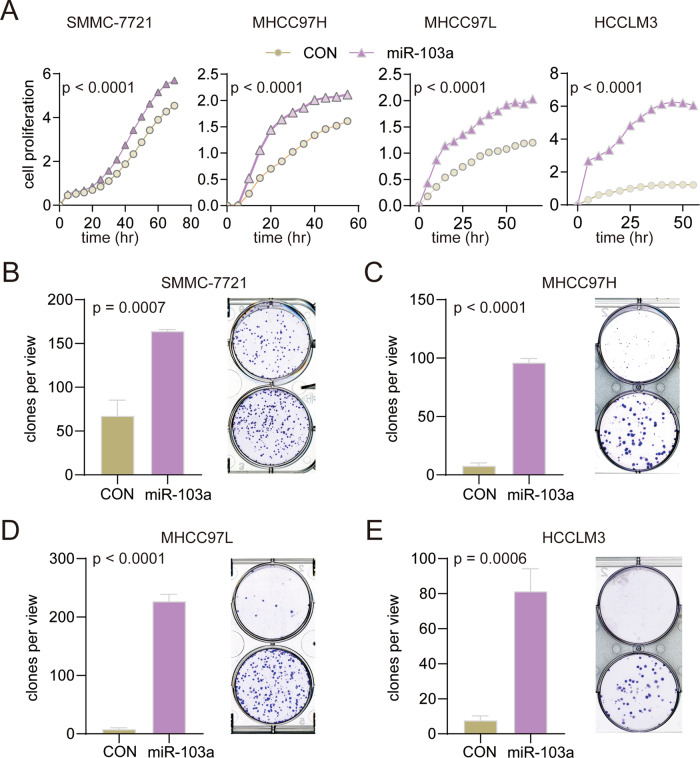
miR-103a overexpression promoted hepatocellular carcinoma cell growth. **A** RTCA was performed to determine the proliferation of SMMC-7721, MHCC97H, MHCC97L and HCCLM3 cells overexpressing miR-103a. **B**–**E** Colony formation assay was performed to examine the colony formation abilities of SMMC-7721, MHCC97H, MHCC97L and HCCLM3 cells overexpressing miR-103a. *p < 0.5, **p < 0.01, ***p < 0.001. All results are representative of at least three independent experiments.

To identify the potential function of miR-103a, a real-time cell analyzer (RTCA) was performed to measure the proliferation rates of miR-103a-overexpressing HCC cells. miR-103a overexpression promoted cell proliferation (Fig. 3A, *p* < 0.0001). Furthermore, the colony formation assay results suggested that miR-103a overexpression remarkably promoted cell colony formation abilities compared with the negative control groups (Fig. 3B–E), and downregulated miR-103 expression in SMMC-7721 inhibited cell proliferation (Fig. S1B).

These data showed that miR-103a promotes cell growth in vitro.

### miR-103a promotes HCC cell migration and invasion in vitro

**Fig. 4:**
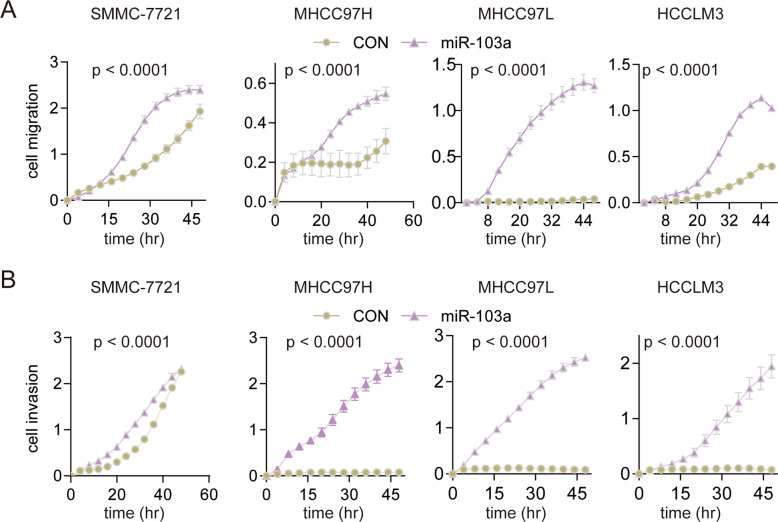
miR-103a promoted the mobility of hepatocellular carcinoma cells. **A** Overexpression of miR-103a promotes HCC cell migration. The migration ability of SMMC-7721, MHCC97H, MHCC97L and HCCLM3 cells overexpressing miR-103a was detected by RTCA. **B** Overexpression of miR-103a promoted HCC cell invasion. The invasion ability of SMMC-7721, MHCC97H, MHCC97L and HCCLM3 cells overexpressing miR-103a was detected by RTCA. All results are representative of at least three independent experiments.

Next, we performed cell migration assays and invasion assays to study the influence of miR-103a on HCC mobility. The data showed that miR-103a overexpression obviously promoted HCC cell migration and invasion relative to the negative control groups (Fig. 4A, B). And downregulated miR-103 expression in SMMC-7721 inhibited cell invasion (Fig. S1C). These data showed that miR-103a overexpression promotes the mobility of HCC cells.

### miR-103a influences glucose metabolism in HCC cells

**Fig. 5:**
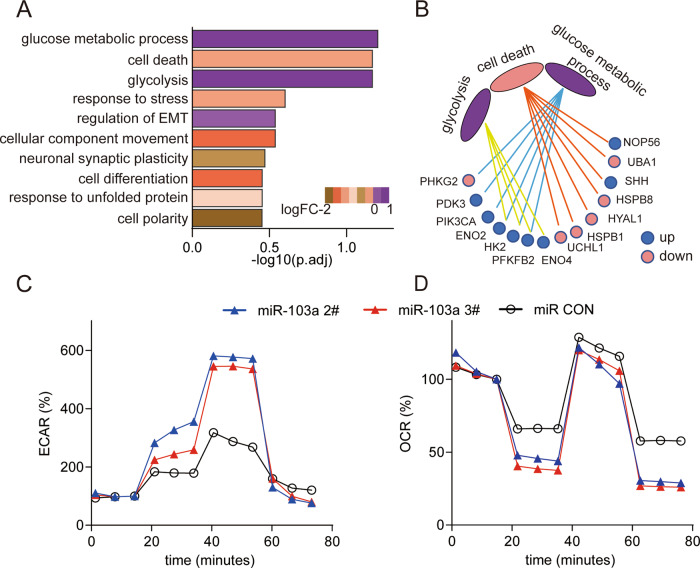
miR-103a influenced glucose metabolism in HCC cells. **A** Functional enrichment analysis revealed that miR-103a participated in glucose metabolic processes, cell death and glycolysis biological processes. **B** Total of 14 genes were matched with these biological functions. **C**, **D**, ECAR and OCR of HCC cells were analysed by a Seahorse XFe 96 Extracellular Flux Analyzer. All results are representative of at least three independent experiments.

To obtain a more comprehensive functional analysis of miR-103, we checked the microRNA expression profile of miR-103a-overexpressing MHCC97L cells by next-generation sequencing. We analyzed the sequencing data by STAR-htseq workflows and then obtained the gene functional enrichment information by the EGSEA r package. In the biological function analysis, we found that the glucose metabolic process, cell death and glycolysis were the top three biological functions (Fig. 5A), with 14 genes participating in these biological functions (Fig. 5B).

To validate the gene functional enrichment information, we assessed the effect of miR-103a overexpression on glycolytic metabolism via a seahorse assay. The results showed that the overexpression of miR-103a enhanced the extracellular acidification rate (ECAR), which reflects the overall glycolytic flux, and decreased the OCR, which is an indicator of mitochondrial respiration (Fig. 5C, D). And downregulated miR-103 expression in SMMC-7721 decreased the ECAR and enhanced the OCR (Fig. S1D/E).

These results revealed that miR-103a mainly influences glucose metabolism in HCC.

### ATP11A and EIF5 are direct targets of miR-103a

**Fig. 6:**
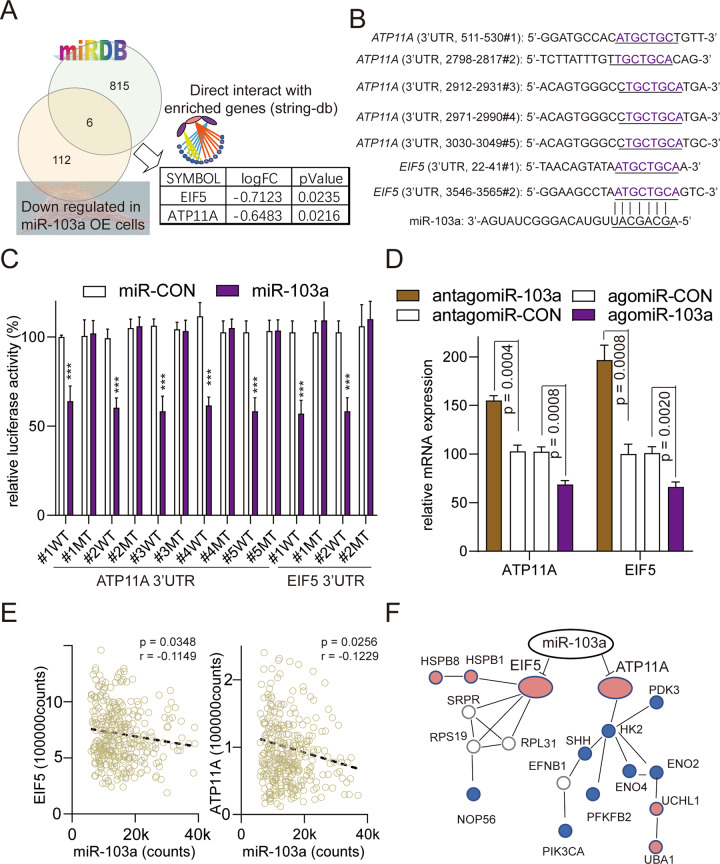
ATP11A and EIF5 were direct targets of miR-103a. **A** Identification of potential miR-103a target genes by microRNA prediction databases and downregulated genes in miR-103a-overexpressing HCC cells. **B** Predicted miR-103a target sequences in the 3′-UTR of ATP11A and EIF5. In addition, the target sequences were mutated. **C** Luciferase reporter activity of WT or MT ATP11A and EIF5 promoters in SMMC-7721 cells transfected with miR-103a mimics and related constructs. **D** MRNA levels of ATP11A and EIF5 in SMMC-7721 cells treated with miR-103a agomiRs or antagomiRs. **E** Correlations between the mRNA expression of miR-103a and ATP11A or EIF5 in TCGA datasets analysed by Pearson’s correlation analysis. **F** miR-protein network analysis using STRINGdb showing that ATP11A and EIF5 were glycolysis-associated target genes of miR-103a. **p < 0.01, ***p < 0.001. All results are representative of at least three independent experiments.

MicroRNAs can repress transcription by binding to complementary sequences in the 3’-UTRs. A target prediction analysis (miRDB) was performed to explore the potential direct targets of miR-103a, and the findings were combined with the results for the downregulated genes in miR-103a-overexpressing MHCC97L cells. Of the 6 candidate genes, ATP11A and EIF5 (Fig. 6A) could interact with the gene enrichment functional network (STRINGdb). Furthermore, the 3′-UTR of ATP11A contains five putative complementary binding sites for miR-103a, and the 3′-UTR of EIF5 contains two putative complementary binding sites for miR-103a (Fig. 6B). To verify that ATP11A and EIF5 are targets of miR-103a, HEK293T cells were transfected with miR-103a agomiR and the luciferase reporter vector harbouring the wild-type or mutant 3’-UTR of ATP11A and EIF5, respectively. The results demonstrated that overexpression of miR-103a significantly decreased the luciferase activity of cells expressing the wild-type but not the mutant 3′-UTR of ATP11A and EIF5 (Fig. 6C). In addition, miR-103a antagomiRs enhanced the mRNA expression of ATP11A and EIF5 in SMMC-7721 cells. In contrast, miR-103a agomiR transfection had the opposite effect on the mRNA of ATP11A and EIF5 (Fig. 6D). Moreover, an analysis using the TCGA database demonstrated that ATP11A and EIF5 had significant negative correlations with miR-103a (ATP11A: *p* = 0.0348, *r* = −0.1149; EIF5: *p* = 0.0256, *r* = −0.1229; Fig. 6E).

Together, these findings revealed that miR-103a could regulate glucose metabolic processes, cell death and glycolysis by directly targeting ATP11A and EIF5.

### Overexpression of miR-103a promotes HCC growth in a xenograft model

**Fig. 7:**
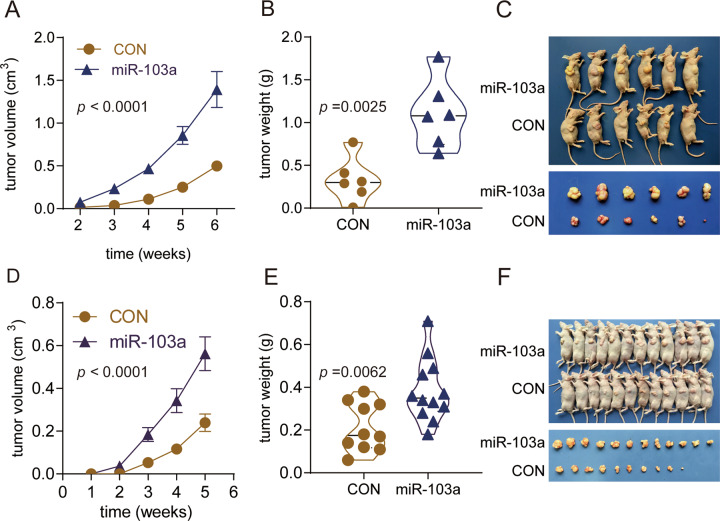
miR-103a promoted cell growth in vivo. miR-103a overexpression promoted SMMC-7721/MHCC97L cell growth in vivo. **A**, **D** Significantly higher tumour volumes were observed in mouse xenografts with HCC cells transfected with the miR-103a mimic than in control xenografts. **B**, **E** Tumour weights were significantly higher in mouse xenografts. **C**, **F** Representative images of xenograft tumours are shown. **p < 0.01, ***p < 0.001.

To further confirm the in vitro findings, we observed the biological roles of miR-103a in vivo. miR-103a overexpression in SMMC-7721 cells increased the tumour volume 2.79-fold relative to that of its control (Fig. 7A) and increased the tumour weight 3.36-fold relative to its control (Fig. 7B), and the representative images are shown in Fig. 7C. MiR-103a overexpression in MHCC97L cells increased the tumour volume 2.35-fold relative to that of the control (Fig. 7D) and increased the tumour weight 1.82-fold relative to that of the control (Fig. 7E), and the representative images are shown in Fig. 7F.

The results demonstrated that after implantation of miR-103a-overexpressing cells, tumour xenografts grew significantly faster than those in the control group.

## Discussion

In this study, we found that miR-103a was a highly expressed and upregulated microRNA in HCC tissues from TCGA. In addition, the high expression of miR-103a was correlated with poor prognosis in HCC patients. Functional experiments revealed that miR-103a overexpression promoted HCC cell growth, migration, invasion and glucose metabolism. Furthermore, the results of bioinformatics methods and dual-luciferase reporter assays showed that miR-103a mainly affected glucose metabolism and cell death by targeting ATP11A and EIF5 directly, thus contributing to HCC cell growth and mobility.

Previous studies have shown that miR-103a plays a critical role in the progression of cancer metastasis. miR-103/107 attenuates miRNA biosynthesis by targeting Dicer, induces the epithelial-to-mesenchymal transition (EMT), and promotes breast cancer cell metastasis [30]. miR-103/107-mediated repression of DAPK and KLF4 promoted metastasis in CRC [31]. miR-103a plays both an oncogenic and tumour-suppressive role in various types of cancers. For example, miR-103 promotes proliferation and metastasis by targeting KLF4 in gastric cancer [32] and regulates triple-negative breast cancer cell migration and invasion by targeting olfactomdin4 [33], while miR-103a functions as a tumour suppressor by directly targeting programmed cell death 10 in NSCLC [34]. A recent study showed that upregulation of serum miR-103 predicts an unfavourable prognosis in patients with colorectal cancer [35]. Taken together, these results suggest that promoting cancer metastasis might be the main function of miR-103a.

miR-103a is also involved in cell growth and metastasis in liver cancer. Previous reports indicated that miR-103a is upregulated in HCC. Moreover, miR-103 promotes HCC cell proliferation and migration in the simulation transition zone of RFA through the PI3K/Akt signalling pathway by targeting PTEN [36], promotes the metastasis and EMT of HCC by directly inhibiting LATS2 [37], and regulates HCC growth by targeting AKAP12 [38]. Moreover, hepatoma cell-secreted exosomal microRNA-103 has been shown to increase vascular permeability and promote metastasis by targeting junction proteins [39]. However, the above studies mainly focused on exploring the effects and mechanisms of miR-103a on HCC cell growth without addressing the role of miR-103a in glucose metabolism in HCC or determining its main function.

In our study, the bioinformatics analysis showed that the EMT, which is the main malignant biological behaviour of miR-103a in the literature, was the fifth most important biological process. Interestingly, miR-103a was mainly involved in the regulation of glucose metabolism and cell death. In the current study, we revealed a novel function of miR-103a in regulating the glucose metabolism phenotypes of HCC.

The liver is a major metabolic organ and a major site of glucose metabolism [40, 41], which is an important process in cancer cell growth [42]. Changes in glucose metabolism have been listed as an important factor that directly contributes to carcinogenesis [43, 44]. However, to the best of our knowledge, no causal association has been established between miR-103a and glucose metabolism in HCC.

In summary, we identified miR-103a as one of the most highly expressed microRNAs in HCC tissues, found that it was upregulated in HCC tissues compared with the controls, and showed that high expression of miR-103a was associated with poor patient prognosis. Moreover, miR-103a overexpression promoted HCC cell growth and mobility, and the functional enrichment analysis showed that miR-103a mainly promoted glucose metabolism and inhibited cell death. We validated this analysis, and the results showed that miR-103a directly influenced glucose metabolism and inhibited cell death via ATP11A and EIF5. Therefore, our study revealed that miR-103a may act as a key mediator in HCC progression.

## Materials and methods

### Cell lines and culture

Four human HCC cell lines (SMMC-7721, MHCC97H, MHCC97L and HCCLM3) and HEK293T cells were purchased from The Cell Bank of Type Culture Collection of the Chinese Academy of Sciences (Shanghai, China). All cell lines were cultured in Dulbecco’s modified Eagle’s medium (DMEM; Gibco, USA) supplemented with 10% foetal bovine serum (FBS; Gibco, USA), 100 units/mL penicillin and 100 μg/mL streptomycin (Gibco, USA) and incubated at 37 °C in humidified incubators containing 5 % CO_2_.

### Clinical samples and TCGA data analysis

Eighty-nine paired samples of tumour tissues (TPs) and their corresponding nontumour tissues (NTs) from patients with HCC and two normal liver tissues were obtained from the surgical specimen archives of Guangxi Medical University, Guangxi Province, China. One of the two normal liver tissue samples was collected from a person who died because of an accident; the other was purchased from Clontech (Palo Alto, CA). The whole transcriptome sequencing (RNA-seq) data of 374 liver TPs and 50 adjacent NTs were obtained from TCGA liver cancer dataset (LIHC) (http://cancergenome.nih.gov). Informed consent was obtained for all human materials, and the institutional ethics review committee of the Shanghai Cancer Institute approved the protocols used in this study.

### RNA extraction and quantitative real-time polymerase chain reaction analysis

Total RNA was extracted from cell lines and tissue samples using a TRIzol kit (Invitrogen, Carlsbad, CA, USA). RNA (1 μg) was reverse-transcribed into cDNA immediately using a Prime-Script RT kit (Takara, Shiga, Japan) following the manufacturer’s instructions. qRT-PCR was carried out with SYBR Premix EX Tag (Takara) on an ABI Prism 7500 fast RT-PCR instrument (Applied Biosystems, Foster City, CA). Each experiment was performed in triplicate. MicroRNA qRT-PCR primers were obtained from RiboBio (Guangzhou, China). The microRNA expression levels were calculated using the delta-delta Ct method, with RNU6B as an internal control. A Ct value of 35 was set as the cut-off value for a nondetected classification. β-actin was used as the internal reference gene for gene expression. Objective CT values were normalized to β-actin, and the 2^−ΔΔCt^ method was used to calculate relative mRNA levels of gene expression. The primer sequences contained in this study were as follows (http://pga.mgh.harvard.edu/primerbank/):

ATP11A: F, 5′-TACCCAGACAACAGGATCGTC-3′ and

R, 5′- AGCCGTCACAGTAATGACAAAG-3′;

EIF5: F, 5′- AGCGTGTCAGACCAGTTCTAT-3′ and

R, 5′- CTGTCTTGATTCCATTGCCTTTG-3′;

β-actin: F, 5′-TTGTTACAGGAAGTCCCTTGCC-3′ and

R, 5′- ATGCTATCACCTCCCCTGTGTG-3′.

### Lentiviral production and transduction

Particles carrying the hsa-pri-miR-103a precursor and its control were purchased from GENECHEM (Shanghai, China). Lentiviral transduction was carried out according to advice from GENECHEM. The high expression was validated by qRT-PCR.

### Proliferation, colony formation, migration and invasion analysis

Cell proliferation, invasion and migration assays were measured with the xCELLigence System’s Real-Time Cell Analyzer (RTCA, Roche/ACEA Biosciences) placed in a humidified incubator and maintained at 37 °C with 95% air/5% CO_2_. This system continuously monitored electrical impedance created by cell adhesion and proliferation in a microelectrode-integrated membrane, and the output was a unitless parameter (cell index). For proliferation assays, 1 × 10^4^ to 3 × 10^4^ cells were seeded into E-plate 16 (ACEA Biosciences) with 200 μL DMEM containing 10% FBS (*n* = 3). The cell index was normalized to the baseline reading at time point 0 and measured every 30 min for 72 h. Migration and invasion assays were performed in 16-well CIM plates (ACEA Biosciences). For migration assays, 1.5 × 10^5^ cells were seeded in triplicate in the upper chamber in a serum-free medium. The upper chamber was then placed on the lower part of the CIM device, which contained DMEM with 10% FBS as a chemoattractant. The cell index was measured every 30 min for 48 h. For the invasion assays, the upper chamber of the CIM-16 plate was initially coated with Matrigel (BD Biosciences, Bedford, MA, USA) diluted in serum-free medium at a ratio of 1:20. The next steps were consistent with those for the migration assay.

For the colony formation assay, 8 × 10^2^ cells were seeded in six-well plates and maintained in DMEM with 5% FBS. After 14 days, the cells were washed with PBS and stained with 0.5% crystal violet (Sigma, USA).

### ECAR and OCR

A Seahorse XF96 Flux Analyzer (Seahorse Bioscience, Billerica, Massachusetts, USA) was used to measure the oxygen consumption rate (OCR) and extracellular acidification rate (ECAR) in lung cancer cells according to the manufacturer’s instructions. Approximately 1 × 10^5^ SMMC-7721 cells per well were seeded into an XF96-well plate and attached overnight. For the assessment of ECAR, cells were incubated with nonbuffered RPMI 1640 under basal conditions, followed by a sequential injection of 10 mM glucose, 1 mM mitochondrial poison (oligomycin, Sigma-Aldrich, Saint Louis, Missouri, USA) and 80 mM glycolysis inhibitor (2-deoxyglucose, 2-DG, Sigma-Aldrich). OCR was assessed under basal conditions and after sequential injection of 1 μM oligomycin, 1 μM fluoro-carbonyl cyanide phenylhydrazone (FCCP) and 2 mM antimycin A and rotenone (Sigma-Aldrich, Saint Louis, Missouri, USA). Both the ECAR and OCR measurements were normalized to the total protein content.

### Target gene prediction of miR-103a and functional enrichment analysis

The bioinformatic online database miRDB was used to predict potential target genes of miR-103a. The consensus results of the miRDB database and RNA-seq were selected for further analysis. Total RNA was isolated from MHCC97L cells using TRIzol reagent (Invitrogen) and purified by an RNeasy Mini Kit (Qiagen). Transcriptome sequencing was conducted using Illumina HiSeq^™^ 2000 by BerryGenomics (Beijing Biotech, China). Gene functional enrichment analyses were performed using EGSEA r-package software, which is a widely used tool for gene functional enrichment. To construct the interaction network, protein-protein interaction data from the STRING database were used. All procedures were conducted according to official protocols and default parameters.

### Transfection of microRNA agomiR and antagomiR

An agomiR is a type of specially labelled and chemically modified double-stranded microRNA that can regulate the biological function of a target gene by mimicking endogenous microRNA. An antagomiR is a type of specifically labelled and chemically modified single-stranded microRNA designed based on the mature microRNA sequence that can inhibit the expression of endogenous microRNA. AgomiR-103a, antagomiR-103a and their respective control materials were procured from RiboBio (Guangzhou, China) and transfected into HCCs and HEK293T cells according to the manufacturer’s protocol. The medium was changed once after 24 h of transfection.

### Luciferase reporter assay

The ATP11A/EIF5 3′-UTR (untranslated region) and mutants were obtained from RiboBio (Guangzhou, China). HEK293T cells were cotransfected with pmiR-103a agomiR or negative controls (NCs). Luciferase activity was measured 24 h after transfection using the Dual-Glo® Luciferase Assay System (Promega) according to the manufacturer’s instructions.

### Tumour xenograft models

A subcutaneous xenograft mouse model was used to assess tumour growth. Animal experiments were approved by the Ethics Committee of Renji Hospital, Shanghai Jiao Tong University School of Medicine. Female nude mice (age, 4–5 weeks; weight, 15–20 g; Institute of Zoology, Chinese Academy of Sciences) were randomly divided into two groups: the miR-103a overexpression group and the CON group (at least 6 per group). A total of 2 × 10^6^ SMMC-7721 cells in 100 µL of DMED without FBS were injected into nude mice. A total of 2 × 10^6^ MHCC97L cells in 100 µL of DMED without FBS were injected into nude mice. The tumour volume was measured by calliper measurements every week and calculated with the formula (length × width^2^)/2.

### Statistical analysis

Data are expressed as the mean ± standard deviation (SD) from at least three independent experiments. Statistical differences between groups were evaluated by Student’s *t* test (paired/unpaired). Two-way analysis of variance (ANOVA) followed by Tukey’s multiple comparisons test was performed to compare significant differences and calculate the *P*-value between the different groups. Pearson correlation tests were performed for the correlation analyses. A survival analysis was performed with the Kaplan–Meier method, and the log-rank test was used for comparisons. Statistical results were analysed using Prism software (GraphPad Software). A probability of 0.05 or less was considered statistically significant.

## Supplementary information

supplemental Figure
