# Supplementary material for: MiR-103a promotes tumour growth and influences glucose metabolism in hepatocellular carcinoma
Source: Cell Death Dis. 2021 Jun 15;12(6):618. doi: 10.1038/s41419-021-03905-3 (PMC8206076; doi:10.1038/s41419-021-03905-3)
Supplement: Supplementary file 1 — supplemental Figure [file 41419_2021_3905_MOESM1_ESM.doc]

Supplementary Figure 1


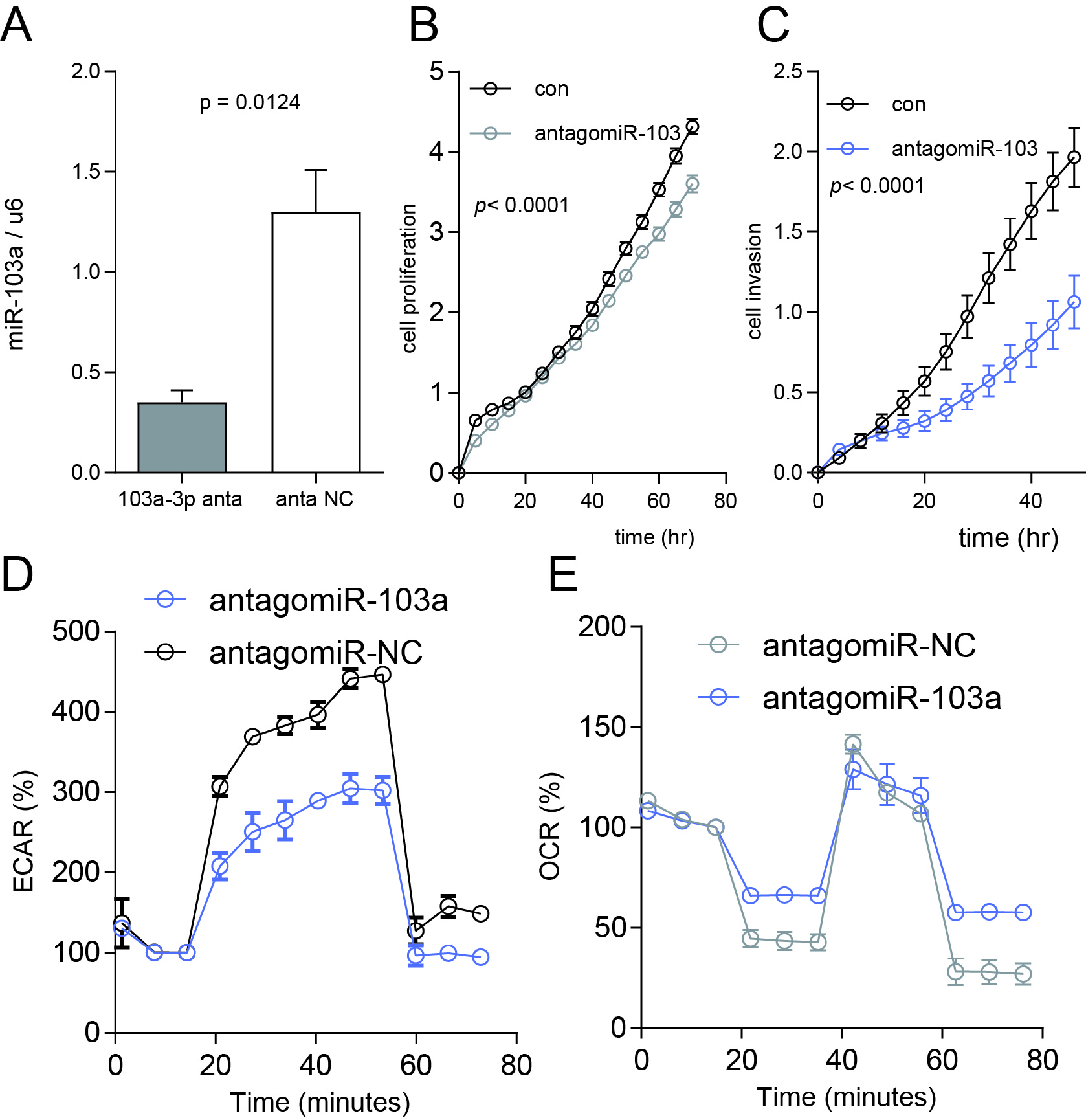


Figure S1. Down-regulated miR-103a inhibited cell proliferation, invasion and glucose metabolism in vitro.

1. Transfected antagomiR-103a and antgomiR-NC into SMMC-7721 cell line. (B) Down-regulated of miR-103a inhibited cell proliferation. (C) Down-regulated of miR-103a inhibited cell invasion. The cell proliferation and invasion ability of miR-103a down-regulated in SMMC-7721 was detected by RTCA. (D,E) Down-regulated of miR-103a inhibited cell ECAR/ OCR ability. The ECAR and OCR of HCC cells were analyzed by Seahorse XFe 96 Extracellular Flux Analyzer. All results were representative of least three independent experiments.

Table s1

Patient characteristic and univariate P value.

| Characteristics | N (%) | *P* |
| --- | --- | --- |
| Age (years) |  |  |
| <50 | 57 (64) | 0.94 |
| ≥50 | 32 (36) |
| Gender |  |  |
| Male | 75 (84.3) | 0.02 |
| Female | 14 (15.7) |
| Size of tumor (diameter/cm) |  |  |
| ≤5 | 25 (28.1) | 0.09 |
| >5 | 64 (71.9) |
| Liver cirrhosis |  |  |
| Absence | 68 (76.4) | 0.76 |
| Presence | 21 (23.6) |
| HBV |  |  |
| Absence | 16 (18) | 0.55 |
| Presence | 73 (82) |
| AFP (ng/mL) |  |  |
| <20 | 27 (30.3) | 0.45 |
| ≥20 | 62 (69.7) |
| Tumor differentiation |  |  |
| Low | 8 (9) | 0.26 |
| Moderate | 66 (74.2) |
| High | 15 (16.9) |
| Tumor embolus |  |  |
| Absence | 54 (60.7) | 0.02 |
| Presence | 35 (39.3) |
| TNM classification |  |  |
| Ⅰ-Ⅱ | 57 (64) | 0.94 |
| Ⅲ-Ⅳ | 32 (36) |
| Edmondson-Steiner stage |  |  |
| Ⅰ-Ⅱ | 45 (50.6) | 0.92 |
| Ⅲ-Ⅳ | 44 (49.4) |
| Vital state |  |  |
| Alive | 19 (21.3) | 0.75 |
| Dead | 70 (78.7) |
